# Multivariate analysis of body morphometric traits in conjunction with performance of reproduction and milk traits in crossbred progeny of Murrah × Jafarabadi buffalo (*Bubalus bubalis*) in North-Eastern Brazil

**DOI:** 10.1371/journal.pone.0231407

**Published:** 2020-04-21

**Authors:** Breno Araújo de Melo, Alberto de Gusmão Couto, Fabiane de Lima Silva, Kuang Hongyu, Filipe Chagas Teodózio de Araújo, Sybelle Georgia Mesquita da Silva, Raisa Rodrigues Santos Rios, Micheline Thais dos Santos, Angelina Bossi Fraga

**Affiliations:** 1 Northeast Network of Biotechnology - RENORBIO, Agricultural Science Center, Federal University of Alagoas, Rio Largo, Alagoas, Brazil; 2 Castanha Grande Farm, São Luiz do Quitunde, Alagoas, Brazil; 3 Departament of Agricultural Science, Federal University of Recôncavo Baiano, Bahia, Brazil; 4 Departament of Statistic, Federal University of Mato Grosso, Cuiabá, Mato Grosso, Brazil; 5 Animal Science Department, State University of Maringá, Maringá, Paraná, Brazil; 6 Animal Science Department, Rural Federal University of Pernambuco, Recife, Pernambuco, Brazil; Tokat Gaziosmanpasa University, TURKEY

## Abstract

We studied the relationship between body morphometric traits, and their underlying association with milk production (MP), lactation length (LL), first calving interval (FCI) and subsequent calving interval (CI) of crossbred progeny of Murrah × Jafarabadi buffalo aiming to assist in selection programs. We carried out principal component analysis (PCA) of the body morphometric traits, which include breast width (BW), thigh width (THW), hip width (HW), rump width (RW), rump length (RL), body depth (BD), body length (BL), height withers (HEW), rear height (RH), shoulder width (SW), thorax width (TW), loin width (LW), distance from the head to ischium (DHI), and thoracic perimeter (TP). We determined the association of morphometric traits with milk/reproduction traits using canonical correlation analysis (CCA). The analysis revealed that the first six PCA accounted for 82.14% of the total observed variation, and the traits THW, HW, TW, LW, RW, HEW, TP, RH, and BW, accounted for almost half (48.00%) of the total variance indicating a higher contribution in body structural conformation. The first canonical function was significant (p<0.05), accounted for 72.46% of the total variance, and the canonical correlation was 0.56, indicating the dependence between both groups of traits. Higher canonical loadings were obtained for LL (0.49), FCI (0.46), BW (-0.71), BL (-0.56), DHI (-0.34), HEW, (-0.38) and TP (-0.50). These traits were most important for the derivation of canonical statistical variables, and presented a higher canonical correlations (r) between the dependent (LL/FCI) and independent (BW, BL, DHI, HEW and TP) groups. The results could suggest that the body morphometric traits THW, HW, TW, LW, RW, HEW, BD, TP, RH, and BW could play important role in body structural composition, indicating a suitable functional type, and aid designing of selection programs for buffalo breeding.

## Introduction

Raw milk and dairy products are important components of the human diet in many regions of the world [1]. The riverine buffalo is the second most common source of milk in several countries [2]. To meet the growing nutritional demand of the global population, it is imperative that breeding programs that meet the demand of animal production to be developed.

Reproductive efficiency and milk production have been used as selection criteria owing to their important role in the financial viability of dairy farms [3]. These criteria follow a body index and standard scores called a functional type, which refers to the association between body conformation and performance of an individual animal. [4,5]. Improvement of milk production in qualitative and quantitative terms may increase the body size of females due to the correlation between those traits. Moreover, selection based on body morphometric traits may lead to an inadvertent selection of undesirable traits. Increased body size, in general, leads to reproductive problems and increases production costs, due to the greater energy demand for a cow’s maintenance. Oltenacu and Broom [6] reported that an increase in body size causes stress and metabolic disorder in animals. Therefore, the selection of animals while considering the association between body morphometric traits and productive/reproductive traits can be effective for obtaining suitable biotypes for each production system. Research has shown the association between biotypes, and productive and reproductive traits [3,6–19]. However, there are limited studies examining functional biotype and their relationship with productive/reproductive in the buffalo. According to Agudelo-Gómez et al. [14], buffalo body morphometric traits can be used to predict the ability for commercial exploitation and when applied in breeding selection programs may contribute to creating an appropriate functional type.

Among the statistical tools applied in animal production, multivariate analysis is an efficient approach used to study compound data for a large number of variables [20]. This technique aims to study several traits simultaneously, which can be difficult to measure or hard for selection programs to understand, especially when there is a negative genetic correlation between them [14]. Multivariate techniques, such as principal component analysis (PCA) and canonical correlation analysis (CCA), have been widely used in animal production study [7,12–17,19–29]. PCA has been used to clarify the structural relationship between different traits and to eliminate redundant traits, thus reducing the sample size of original set of variables [30,31]. CCA facilitates a linear combination for each set of variables in a study, so that the correlation between these two sets would be maximized [32]. This technique can assist in the analysis of several metric traits and, besides it may indicate the factors most relevant to the set of variables studied. The present study was carried out to investigate clarify the structural relationship between different body morphometric traits aiming to eliminate redundant traits on body composition, and their underlying association with milk/reproductive traits in crossbred progeny of Murrah × Jafarabadi buffalo by multivariate analysis to aid buffalo selection programs.

## Materials and method

### Experimental animals and management

The present investigation was carried out at the Agricultural Science Center of the Federal University of Alagoas, Rio Largo, Brazil, and was approved by the Ethics Commission for animal experimentation of this university. The investigation contained the database on milk/reproductive traits, and body morphometric traits of 99 crossbred progeny of Murrah × Jafarabadi buffalo, belonging to a farm located in northeastern Brazil. The geographic coordinates of the study location are 9°10′06″S and 35°33′40″W, with a rainy, tropical climate, according to Köppen classification and at approximately 10 m altitude.

Lactating buffaloes were milked twice a day, in the morning and afternoon, by mechanical milking. The animals were kept in a semi-intensive system providing feeding on *Brachiaria humidícula* pasture, and crushed sugarcane (*Saccharum officinarum*) enriched with urea (10g of urea/kg of sugarcane). In addition, mineral supplementation and balanced rations were provided (the buffalo with production between 5 to 7.5 L milk/day received 1 kg of ration/day; buffalo with production between 8 to 10.5 L milk/day received 2 kg of ration/day). These buffaloes reproduced in the natural breeding season with estrus synchronization.

### Traits investigated

The studied traits were calving interval (CI), period between two consecutive calving; first calving interval (FCI), period between the first and second calving; milk production/lactation (MP); and lactation length (LL). The body morphometrics were breast width (BW), thigh width (THW), hip width (HW), rump width (RW), rump length (RL), body depth (BD), body length (BL), height withers (HEW), rear height (RH), shoulder width (SW), thorax width (TW), loin width (LW), distance from the head to the ischium (DHI) and thoracic perimeter (TP).

The body morphometric traits were measured in centimeters and always on the left side of the animal before morning milking, using measuring tape and a pachymeter as follows: BW, the width between one side of the breast to the other; THW, the distance between the external angles of the femoral joints; HW, the length from one hip to the other; RW, the distance between the ischia; RL, the distance between the ileum and ischium; BD, the distance between the last thoracic vertebra and the umbilical region; BL, the diagonal length of the body between the bottom tip of the scapula until the tip of the ischium; HEW, the distance between the withers and the distal extremity of the front limb; RH, the distance between the sacral tuberosity and the distal extremity of the back limb; SW, the distance between the both scapula; TW, the distance between the two lateral lines passing through the dorsal angles of the scapula; LW, the distance between the thoracic vertebra and lumbar vertebra; DHI, the distance between the occipital protuberance until the tuberosity of the ischium; and TP, the contour of the thorax passing behind the front limbs and returning perpendicularly by the withers [33].

### Statistical analysis

Descriptive analyses and underlying correlations were performed for all the variables, considering the last performance of MP, LL and CI for each female buffalo. As body traits were measured at any physiological stage (pregnant or not), these records were grouped according to physiological stages: category 1—period between conception and 90 days after conception; category 2—period between 90 and 180 days after conception; category 3—period between 180 and 270 days after conception and category 4—period between calving and conception. After the identification of all possible sources of variation, a preliminary analysis including all of these effects was performed (calving order and physiological stages) aiming for the adjustments of these factors. However, the preliminary results showed that these effects were not significant (p>0.05) and, therefore, were not considered in any further analyses.

### Principal component analysis (PCA)

The principal components are calculated through linear combinations of the original variables with eigenvectors (body morphometric of 99 buffaloes). The absolute value of an eigenvector determines the importance of the traits in a principal component. Each eigenvector is calculated from an eigenvalue of the correlation matrix of the data, where the eigenvalues are related to the variance of each principal component [34]. PCA consists of transforming the set of variables (X_i1_, X_i2_, …, X_ip_) into a new set (Y_i1_, Y_i2_, …, Y_ip_), which are linear functions of the **X**_p_ and independent of each other. Therefore, **Y**_i1_ is a principal component, calculated by linear combinations of the original variables with eigenvectors (S1 File).

The PCA was used to study the body conformation of the buffalos considering 14 body measurement traits. The principal components with eigenvalues greater than 0.70 (λ_i_>0.7) were considered as were those with the highest percentage of variance according to Jolliffe [35,36]. The correlations between each trait, and the correlations between traits and the principal components were estimated. These analyses were performed using procedure PCA and PRCOMP, FactoMineR library [37] available from R software version 3.6.2 [38].

### Canonical correlation analysis (CCA)

The CCA aimed to estimate the maximum correlation between linear combinations of traits of group I and group II, as well as to estimate the respective weighting coefficients of the traits in each linear combination. Let **X**' and **Y**' be two groups of variables **X** and **Y**, defined as: X′ = [*X*_1_ *X*_2_ … *X*_3_] is the vector that measures p traits constituting group I; *Y*′ = [*Y*_1_ *Y*_2_ … *Y*_3_] is the vector that measures q traits constituting group II. As **X**_1_ and **Y**_1_ are the linear combinations of the variables belonging to groups I and II, respectively [39]:

$$X_{1}=a_{1}x_{1}+a_{2}x_{2}+\ldots +a_{p}x_{p}$$ (1)

$$Y_{1}=b_{1}y_{1}+b_{2}y_{2}+\ldots +b_{q}y_{q}$$ (2)

In order that the correlation between (**X**, **Y**) be maximized, the first canonical function, **X**_1_ and **Y**_1_ will be obtained:

$$r_{1}=\frac{Côv(X_{1}{,Y}_{1})}{\sqrt{\hat{V}(X_{1})\hat{V}(Y_{1})}}$$ (3)

If **X** be the matrix m × p and **Y** be the matrix n × q, *C* = *cov* (*X*, *Y*), sharing the matrix C in four parts, we have:

$$\text{C}=\left[\begin{array}{cc} \sum_{pxp}11 & \sum_{pxq}12\\ \sum_{qxq}21 & \sum_{qxp}22 \end{array}\right]$$ (4)

Therefore, the CCA produced a set of new variables, called the canonical functions (CF), which are linear combinations of the original variables, as reported in the following equation:

$$\text{CF}=d_{1}X_{1}+d_{2}X_{2}+d_{3}X_{3}+\cdots +d_{n}X_{n}$$ (5)

where d_n_ are the canonical coefficients that indicate the contribution of each variable in the composition of CF, and **X** are the scores of the *n* original variables.

In this study, CCA was performed to verify the relationship between one group of dependent variables from milk/reproductive traits (MP, LL, CI, and FCI) and a second group of independent variables (body morphometric traits). Although 14 body morphometrics were available, we included just nine variables (BW, TW, RW, RL, BD, BL, DHI, HEW, and TP) to perform the CCA. These variables were preliminarily chosen, because they have shown a higher contribution in shared variance with milk/reproductive traits.

In this study, the variance inflation factors (VIF) for milk/reproductive and body measurement traits were less than 10 (VIF<10) for all of them, therefore no multicollinearity existed between the two variable groups (S2 File). The multicollinearity compromises the accuracy of the CCA due to the existence of collinear variables [40].

Then, the simple linear correlation (r) and canonical correlation (R^2^) were estimated between the two trait groups using the multivariate significant Wilks’ Lambda test (approximation of the distribution F) to evaluate the significance of canonical roots jointly. The canonical loadings, correlation between the original variables and their canonical variables (dependent or independent), were obtained. Furthermore, the cross-loadings were also estimated to assess the correlation between an original variable (dependent or independent) and canonical variable of another group. The same was performed for squared loading, obtained from the square of the canonical loading, which explains the amount of shared variance between dependent and independent variables, and their respective canonical statistical variables. Subsequently, the cross-loadings were obtained, in the aim of expressing the correlation between each dependent or independent variable and canonical statistical variable opposite. The squared cross-loadings were obtained to estimate the percentage of shared explained variance between observed variables and canonical variables of the other group.

CCA redundancy indices were calculated by multiplication of the squared loadings average with canonical R^2^. The CCA was subjected to a validation method aiming to ensure the results were not specific. Thus, a sensitivity test was performed, in order to remove any independent variables from the analysis. This test search did not change the values of the canonical loadings, p-value, redundancy index, correlation (r) and canonical R^2^. CCA was developed using the procedure CCA, statistical package YACCA [41] available from R software 3.6.2 [38].

## Results

### Descriptive statistics

The mean values (standard deviation) of the MP, LL, CI and FCI of the buffaloes were 1,656 (609.32) kg/lactation, 257.20 (56.05) days, 441.10 (47.54) days and 489.20 (117.00) days, respectively. The mean ± SE estimated (S3 File) for body morphometric traits (cm) were: BW (43.72 ± 5.21), SW (33.20 ± 4.44), TW (35.38 ± 4.44), LW (37.72 ± 4.75), THW (51.43 ± 5.39), HW (40,03 ± 5.61), RW (25.44 ± 4.21), RL (39.86 ± 4.06), BD (71.77 ± 6.25), BL (143.33 ± 8.53), DHI (182.80 ± 9.40), HEW (130.50 ± 5.03), RH (134.00 4.78), and TP (201.30 ± 8.10).

The higher linear correlations (p<0.01) were obtained for FCI/CI (0.53) and MP/LL (0.47). The correlations (p < 0.01) between milk/reproductive traits were low: MP/FCI (0.00); MP/CI (0.06); LL/FCI (0.02) and LL/CI (0.17). The linear correlations between the body morphometric traits varied from -0.01 to 0.74, were significant (p<0.01) and the highest correlations (p<0.01) between were: SW/TW (0.74); THW/HW (0.69); RH/TP (0.68); HW/RW (0.66); HEW/RH (0.65) and THW/RW (0.64).

### Principal component analysis

**Fig 1:**
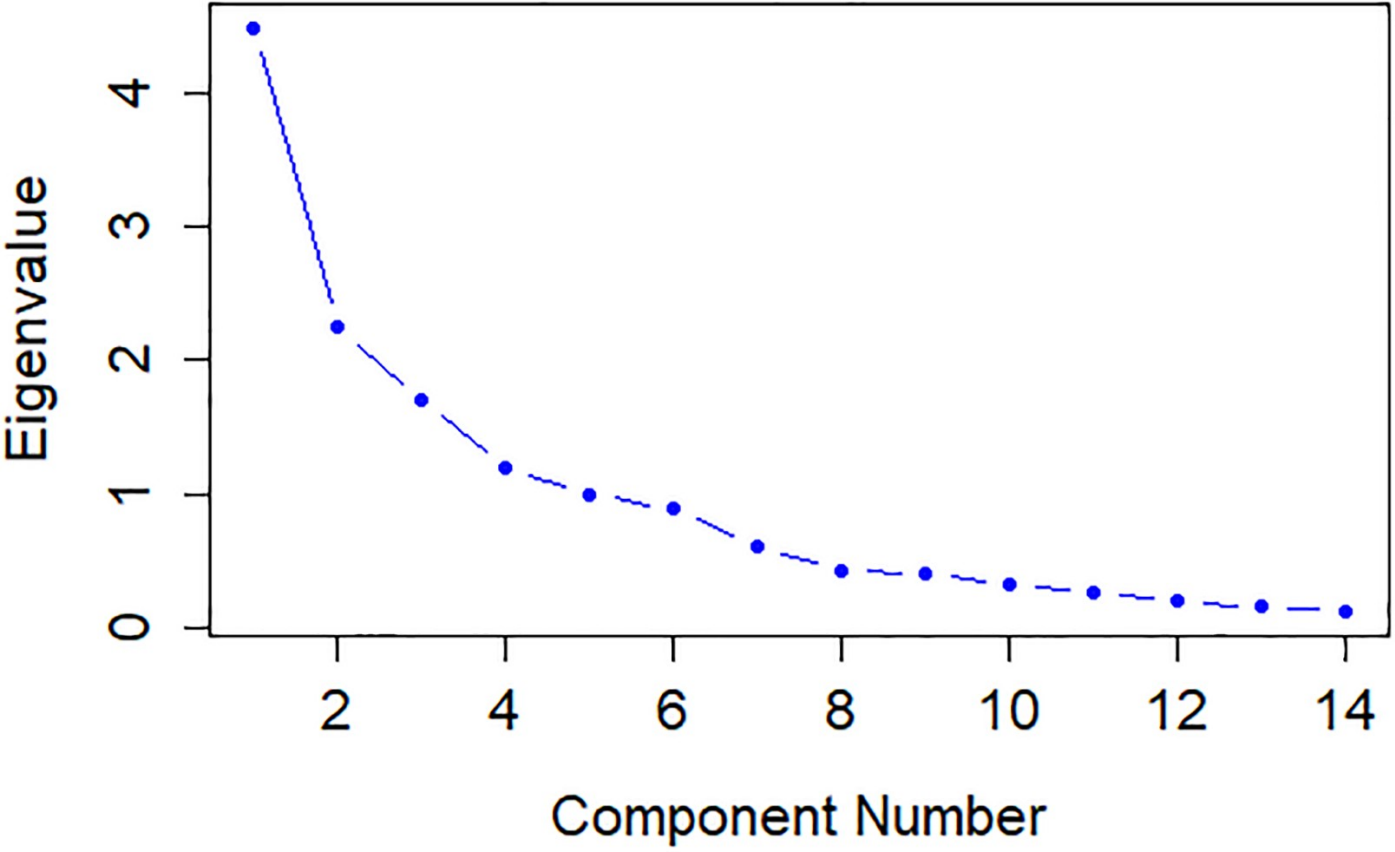
Principal components and eigenvalues for body morphometric traits of crossbred progeny of Murrah × Jafarabadi buffalo.

The eigenvalues (λ_i_) and percentages of the explained variance and accumulated variance for body morphometric traits are presented in Table 1. The results show that the first six components accounted for 82.14% of the total variance, of which the eigenvalues were larger than 0.70 (λ>0.7), Fig 1. The remaining eight PCs had lower variance (S4 File). The first (PC1), second (PC2), third (PC3) and fourth (PC4) principal components accounted for 32.00%, 16.00%, 12.10% and 8.60% of the total variance, respectively, while the remaining PCs jointly accounted for 31.32% of the total variation.

**Table 1:** Principal components (PC), eigenvalues (λi), percentage of explained variance (%VAR), and cumulative variance (%CV) of body morphometric traits of crossbred progeny of Murrah × Jafarabadi buffalo.

PC	λ_i_	%VAR	% CV
PC1	4.4803	0.3200	0.3200
PC2	2.2395	0.1600	0.4800
PC3	1.6931	0.1209	0.6009
PC4	1.2025	0.0859	0.6868
PC5	0.9965	0.0712	0.7580
PC6	0.8880	0.0634	0.8214
PC7	0.6005	0.0429	0.8643
PC8	0.4278	0.0306	0.8949
PC9	0.4046	0.0289	0.9238
PC10	0.3292	0.0235	0.9473
PC11	0.2622	0.0187	0.9660
PC12	0.1981	0.0142	0.9802
PC13	0.1560	0.0111	0.9913
PC14	0.1216	0.0087	1.0000

**Fig 2:**
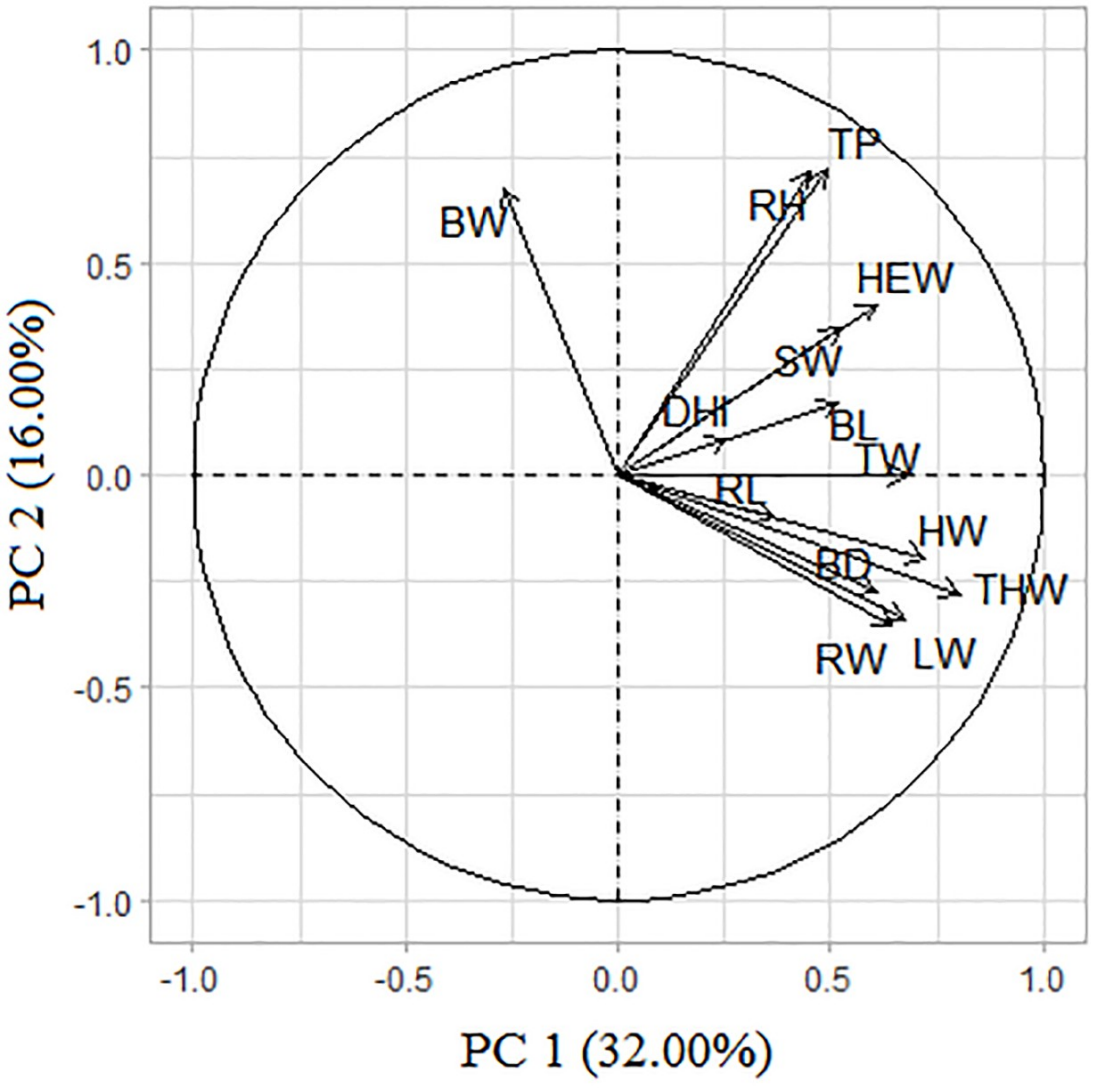
Eigenvectors of body morphometric traits on the first two principal components of crossbred progeny of Murrah × Jafarabadi buffalo. Thigh width (THW), hip width (HW), thorax width (TW), loin width (LW), rump width (RW), height withers (HEW), body depth (BD), shoulder width (SW), body length (BL), thoracic perimeter (TP), rear height (RH), rump length (RL), breast width (BW), and distance from the head to ischium (DHI).

The correlation between the original variables and the PCs were calculated in order to understand the importance of each trait in the composition of PCs. The correlation between PCs and the traits studied, and their weightings are shown in Table 2. Therefore, PCs can be expressed in equations (S5 File), which are linear combinations of the original variable and weighting coefficients. In order to aid the visual inspection of strength and direction of the eigenvectors on the two first PCs, the traits were presented in cartesian plan (Fig 2), while further figures were presented in S6 File.

**Table 2:** Weighting coefficients, correlations between principal components and body morphometric traits of crossbred buffalo.

Traits^1^	Correlations						Weighting coefficients					
	PC1	PC2	PC3	PC4	PC5	PC6	PC1	PC2	PC3	PC4	PC5	PC6
THW	0.80	-0.28	0.00	0.23	-0.15	-0.26	-0.38	-0.19	0.00	-0.21	-0.15	-0.27
HW	0.72	-0.19	-0.15	0.37	-0.29	0.23	-0.34	0.13	-0.12	-0.34	-0.29	0.25
TW	0.68	0.00	-0.55	0.01	0.36	-0.02	-0.32	0.00	-0.42	-0.01	0.36	-0.02
LW	0.67	-0.34	-0.15	-0.28	0.11	0.19	-0.32	0.23	-0.12	0.25	0.11	0.20
RW	0.64	-0.35	-0.14	0.16	-0.48	0.08	-0.30	0.23	0.11	0.14	0.48	-0.09
HEW	0.61	0.40	0.21	-0.26	-0.35	-0.22	-0.29	-0.27	-0.16	-0.24	0.35	0.23
BD	0.60	-0.27	0.50	-0.24	0.24	-0.06	-0.29	0.18	-0.38	-0.22	-0.24	0.07
SW	0.52	0.34	-0.56	0.06	0.40	0.06	-0.25	-0.23	0.43	0.05	-0.40	-0.07
BL	0.51	0.17	0.50	0.21	0.21	-0.34	-0.24	-0.11	-0.38	0.19	-0.21	0.37
TP	0.49	0.72	-0.05	-0.06	-0.05	0.18	-0.23	-0.48	0.04	-0.05	0.05	-0.20
RH	0.45	0.72	0.05	-0.30	-0.11	-0.14	-0.21	-0.48	-0.04	-0.28	0.11	0.15
RL	0.37	-0.10	0.53	-0.27	0.11	0.63	-0.17	0.07	-0.40	-0.25	-0.11	-0.67
BW	-0.27	0.67	0.06	0.39	-0.14	0.30	0.12	-0.45	0.04	-0.35	0.14	-0.32
DHI	0.25	0.08	0.43	0.64	0.29	0.04	-0.12	-0.06	-0.33	0.58	-0.29	-0.05

^1^Thigh width (THW), hip width (HW), thorax width (TW), loin width (LW), rump width (RW), height withers (HEW), body depth (BD), shoulder width (SW), body length (BL), thoracic perimeter (TP), rear height (RH), rump length (RL), breast width (BW), and distance from the head to ischium (DHI).

### Canonical correlation analysis

The four canonical functions (CF) from two variable groups (milk/reproductive and body morphometric traits), multivariate Wilks’ Lambda test, F test and distribution approximation (Fa) are presented in Table 3. Only the first canonical function (CF1) was significant (p<0.05), with canonical correlation coefficient (R^2^ = 0.32) and canonical correlation (r = 0.56). In addition, the CF1 explained 72.46% of the total variance, of which the eigenvalue (λ_i_) was 47,34% (S7 File).

**Table 3:** Wilks’ Lambda multivariate test for canonical functions from milk/reproductive and body morphometric traits of crossbred progeny of Murrah × Jafarabadi buffalo.

	Wilks’ Lambda test					
CF	r	R^2^	Wilks	df	F_a_	p-value
1	0.5668	0.3213	0.5703	36	1.4552	0.0495*
2	0.2874	0.0826	0.8403	24	0.6517	0.8942^ns^
3	0.2323	0.0540	0.9160	14	0.5642	0.8900^ns^
4	0.1784	0.0329	0.9682	6	0.4878	0.8160^ns^

Canonical functions (CF), canonical correlation (r), canonical correlation coefficient (R^2^), percent variance in dependent variables not explained by differences in levels of the independent variable (Wilks), degree of freedom regarding of the treatments (df), approximate F statistics (F_a_),

* (*p* < 0.05),

^ns^ not significant.

In this study, the higher canonical loadings and cross-loadings of CF1 between the milk and reproductive traits (dependent variables) were LL and FCI. Among the body morphometric traits (independent variables), the higher canonical loadings were BW, BL, DHI, HEW, and TP, Table 4. Therefore, these variables were considered the most important in the derivation of canonical statistical variables.

**Table 4:** Canonical loadings and cross-loadings between milk/reproduction traits and body morphometric traits of crossbred progeny of Murrah × Jafarabadi buffalo.

Variables^1^	Canonical loadings	Cross-loadings
Milk/reproductive traits		
MP	-0.2535	-0.1437
LL	0.4942	0.2802
CI	0.2322	0.1316
FCI	-0.4669	-0.2646
Body morphometric traits		
BW	-0.7874	-0.4463
TW	0.1574	0.0892
RW	0.1810	0.1026
RL	-0.0813	-0.0461
BD	0.0162	0.0092
BL	-0.5636	-0.3194
DHI	-0.3495	-0.1981
HEW	-0.3858	-0.2187
TP	-0.5050	-0.2863

^1^Milk production (MP), lactation length (LL), calving interval (CI), first calving interval (FCI), breast width (BW), thorax width (TW), rump width (RW), rump length (RL), body depth (BD), body length (BL), distance from the head to the ischium (DHI), height withers (HEW), and thoracic perimeter (TP).

**Fig 3:**
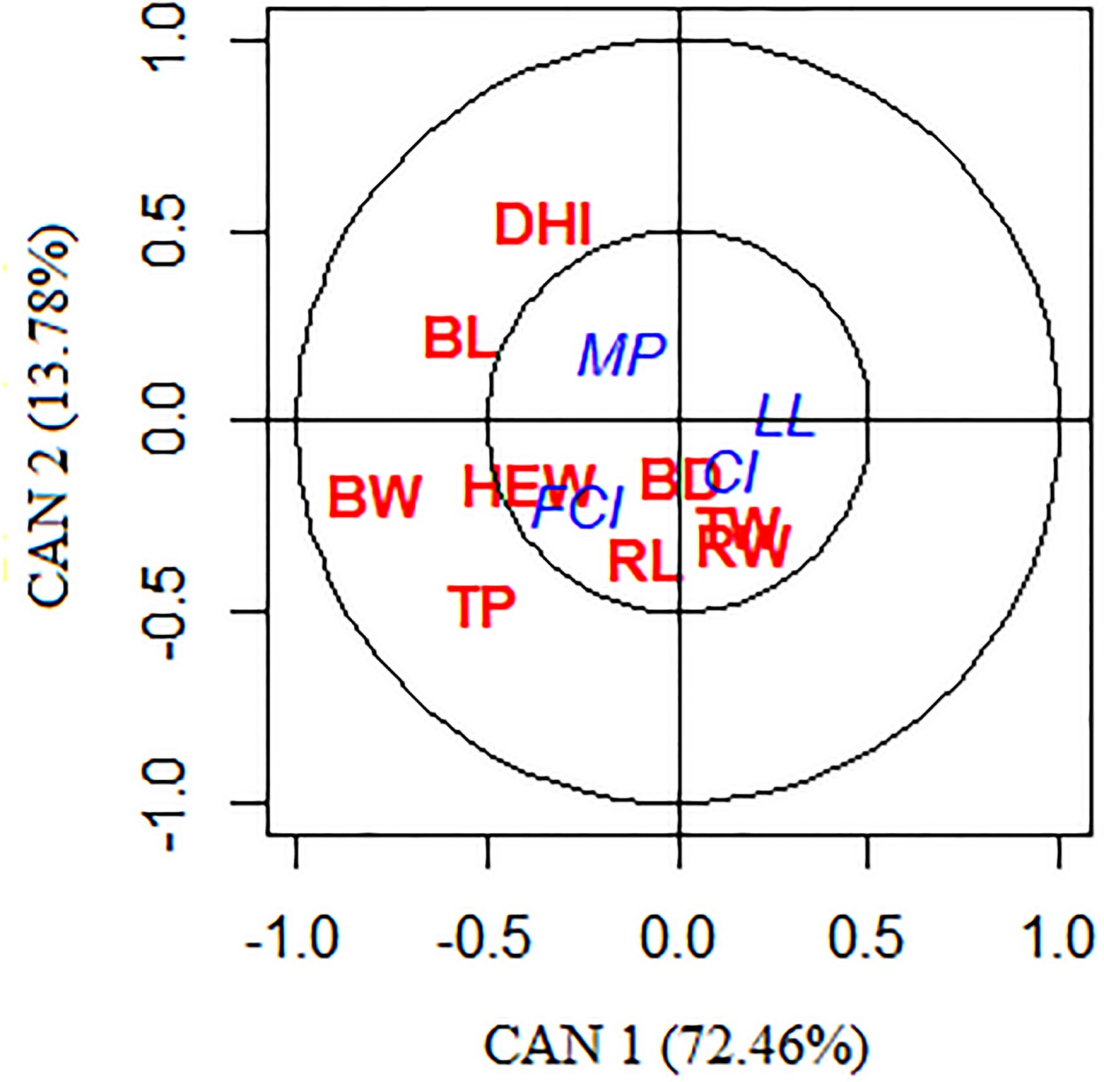
Canonical correlation between milk/reproductive traits and body morphometric traits of crossbred progeny of Murrah × Jafarabadi buffalo. Milk production (MP), lactation length (LL), calving interval (CI), first calving interval (FCI), breast width (BW), thorax width (TW), rump width (RW), rump length (RL), body depth (BD), body length (BL), distance from the head to the ischium (DHI), height withers (HEW), and thoracic perimeter (TP).

The higher cross-loadings between the variables were LL and FCI (as dependent) and BW, BL, DHI, HEW, and TP (as independent), as seen in Table 4. The cross-loading of the FCI showed that this trait was positively correlated with BW, BL, DHI, HEW, and TP, being higher for BW. The variables MP, CI, TW, RW, RL and BD presented low cross-loadings (Fig 3).

Shared explained variance by the canonical statistical variable, dependent and independent, were expressed by squared loadings, squared cross-loadings, and CCA redundancy index, as shown in Table 5. The squared loadings of CF1 had shown that the amount of the shared explained variance by the dependent canonical statistical variable (milk and reproductive traits) was 14.51%. While, the amount of shared variance in the body morphometric traits explained by the independent canonical statistical variable was 16.98%.

**Table 5:** Squared loadings, squared cross-loadings, and CCA redundancy index (%) of milk/reproduction and body morphometric traits of crossbred progeny of Murrah × Jafarabadi buffalo.

Variables^1^	Squared loadings	Squared cross-loadings	CCA redundancy index
Milk/reproductive traits			4.66
MP	0.0643	0.0207	
LL	0.2443	0.0785	
CI	0.0540	0.0173	
FCI	0.2180	0.0700	
Mean	0.1451	0.0466	
Body morphometric traits			5.45
BW	0.6199	0.1992	
TW	0.0248	0.0080	
RW	0.0328	0.0105	
RL	0.0066	0.0021	
BD	0.0003	0.0001	
BL	0.3176	0.1020	
DHI	0.1222	0.0393	
HEW	0.1489	0.0478	
TP	0.2551	0.0820	
Mean	0.1689	0.0545	

^1^Milk production (MP), lactation length (LL), calving interval (CI), first calving interval (FCI), breast width (BW), thorax width (TW), rump width (RW), rump length (RL), body depth (BD), body length (BL), height withers (HEW), distance from the head to the ischium (DHI) and thoracic perimeter (TP).

The result of CCA redundancy index showed that 4.66% of variance of the dependent variables (milk/reproduction) was explained by the independent canonical statistical variables. While, 5.45% of variance of the independent variables (body morphometrics) was explained by the dependent canonical statistical variables. This result could confirm that both the groups were dependent and there was an association between each of them.

The sensitivity test did not indicate any relevant differences between the CCA presented in this study and CCA with the elimination of any of the independent variables (Table 6). The results for the canonical loadings, cross-loadings, canonical correlations (r), canonical correlation coefficients (R^2^), CCA redundancy indexes, and p-value could not demonstrate any differences between the two CCA (Table 6).

**Table 6:** Canonical loadings, cross-loadings, CCA redundancy index, canonical correlation (r), canonical correlation coefficient (R^2^), and p-value after the sensitivity test for the elimination of the thoracic perimeter (TP) for the first canonical function.

Variables^1^	Canonical loadings^2^	Canonical loadings^3^	Cross-loadings^2^	Cross-loadings^3^
Dependents variables				
MP	-0.2535	-0.2625	-0.1437	-0.1483
LL	0.4942	0.4902	0.2802	0.2769
CI	0.2322	0.2494	0.1316	0.1409
FCI	-0.4669	-0.4505	-0.2646	-0.2544
Independents variables				
BW	-0.7874	-0.7897	-0.4463	-0.4461
TW	0.1574	0.1614	0.0892	0.0911
RW	0.1810	0.1838	0.1026	0.1038
RL	-0.0813	-0.0776	-0.0461	-0.0434
BD	0.0162	0.01874	0.0092	0.0106
BL	-0.5636	-0.5651	-0.3194	-0.3192
DHI	-0.3495	-0.3566	-0.1981	-0.2014
HEW	-0.3858	-0.3855	-0.2187	-0.2177
TP (Eliminate)	-0.5050	Eliminate	-0.2863	Eliminate
CCA redundancy index				
Dependents variables	4.66^2^	4.58^3^		
Independent variables	5.45^2^	5.12^3^		
Wilks’ Lambda test				
r	0.5668^2^	0.5648^3^		
R^2^	0.3213^2^	0.3190^3^		
p-value	0.0459*^,^^2^	0.0238*^,^^3^		

^1^Milk production (MP), lactation length (LL), calving interval (CI), first calving interval (FCI), breast width (BW), thorax width (TW), rump width (RW), rump length (RL), body depth (BD), body length (BL), distance from the head to the ischium (DHI), height withers (HEW), and thoracic perimeter (TP) (removed).

^2^Results before of sensitivity test.

^3^Results after the sensitivity test.

* (p < 0.05);

^ns^, not significant.

## Discussion

The average MP (1,656 kg/lactation) could be compared with the previous reports of 1,496 to 2,130 kg/lactation [27,42–49]. The attributed differences might be due to genetic or environmental factors, such as management, genetic composition, and regional climate factors. The LL average (257.20 days) could be compared to reported values from 258 to 301 days of lactation [43,44,46,48,49]. The calving interval mean was 441.10 days, while values between 361 and 451 days have been reported [27,43–45,50]. In addition, the mean of FCI (489 days) could be compared with earlier report of 438 days [13]. This trait accounting the first reproductive cycle of a female, being vital for selection programs.

The BW, SW, and TW account for muscle development and rib arching, which reflect on breathing ability. The present THW average could be compared with earlier reports in Murrah [13,20], Gorji [25] and Nili Ravi [51]. According to de Melo et al [33], wider hips provide better female reproductive ability, in regards to fetal development and natural calving. According to Peixoto et al. [52], the rump should be broad, long and with a good muscular structure. A suitable shaping in this region provides a good angularity of the sacral region, allowing the rump to be slightly inclined and horizontal. This region is important for reproduction ability, since includes the reproductive organs, especially, the womb. Animals with a higher rump width could present a higher uterine development, while animals with a lower rump width are more prone to have difficult calving. The earlier reports corroborated the present estimates for RW [25], RL [51] and BL [51, 53–55].

The present TP average was higher than the available report [13]. This trait could be associated with intake/digestive and breathing ability since it represents the thoracic capacity. With regards to stature traits, the average for HEW (130.05 cm) and RH (130.80 cm) indicated that the female buffalo presented body linearity. Khan et al. [55] did not present body linearity in Azikhele buffalo for HEW (131.35 cm) and RH (123.41cm). The HEW average was corroborated by Mirza et al. [56]. The higher correlation between SW/TW (0.74) could indicate that large shoulders indicate a wider thorax allowing higher breathing ability, which is an important factor for animal metabolism [33]. The estimated correlations of THW/HW (0.69), RH/TP (0.68), HEW/RH (0.65) and HW/RW (0.66) indicate that these body traits are associated with body harmony in the female buffalo. The present correlations of BL/HEW (0.37) and RW/RL (0.13) were lower than the reported correlations of BL/HEW (0.66) and RW/RL (0.89) [54]. Present correlation of THW/RW (0.64) was also lower than the report of 0.91 [55]. Regards to THW/BL (0.47), it was lower than 0.57 [55], and higher than 0.44 [18]. According to Dhillod et al. [18], bovine body traits have been intensively studied, in various parts of the world. However, this approach has been less studied in buffalo. It is crucial to carry out more research to clarify the relationships between body morphometric of buffalo.

PCA has been widely used in animal production in order to understand the relationship between several traits that are of economic interest, and to understand the contribution of each trait in each PC [13–20,22–27,57,58]. In this study, we verified that the first six PCs explained 82.14% of the total variance, and the importance of each body measurement in the composition of these six PCs were determined by the correlations between the original variables and PCs, such as their weighting coefficients (Table 2). The first and second, PCs, which accounted for 48.00% of the total variance, presented higher loadings for thigh width (THW), hip width (HW), thorax width (TW), loin width (LW), rump width (RW), height withers (HEW), body depth (BD), thoracic perimeter (TP), rear height (RH), and breast width (BW). These traits jointly accounted the reproduction ability (THW, HW, LW, RW, and RH), as well as, breath and intake/digestive capacity (HEW, TW, BD, TP and BW). The incidence of eigenvectors on each PC indicated the strength and direction of each trait on PC1 and PC2 (Fig 2). The most important traits for PC1 (THW, HW, TW, LW, RW, HEW and BD), are represented by the higher arrows (Fig 2). Similarly, the most important traits for PC2 such as TP, RH and BW, are represented by the higher arrows (Fig 2). In a similar study, Vohra et al. [25] analyzed 13 morphometric traits in the female buffalo and obtained four PCs explaining 70.90% of the total variance. According to that study, the first component (with 31.5% of variance) described the bulk of the body conformation, suggesting that the PC1 could be used in evaluation and comparison of body conformation in the female buffalo, and to distinguish early and late animals.

The first canonical function (CF1) explains 72.46% of data variance (S5 File), and the maximum and significant canonical correlation (r = 0.57) between the two sets of variables, Table 3. This result could suggest for positive correlation between dependent and independent variables. Therefore, the groups of milk/reproductive traits and body morphometric are dependents, and body morphometric should be considered during the choice of female buffalo, when the aim is to improve the milk and reproductive ability. Canonical correlation analysis has been used in animal production in order to establish its relationship with economic interest traits. Thomas and Chakravarty [7], examined the association between breeding efficiency/breeding value for milk and growth/reproduction traits in female Murrah buffalo, and obtained a positive and significant canonical correlation (r = 0.97) between these two groups of variables, showing the dependency between them. In sheep, Çankaya and Kayaalp [28], showed significant first canonical function and canonical correlation of 0.93 between body morphometric traits and body weights. In swine, Barbosa et al. [21], showed that three (r = 0.41, r = 0.34, r = 0.33) canonical functions for meat quality and carcass traits were significant. In quail, Ribeiro et al. [29], reported significant canonical function (r = 0.34) for egg production and reproduction traits.

In this study, with regard to canonical function 1, the higher (absolute value) canonical loadings were between LL and FCI (dependent variable) and BW, BL, DHI, HEW, and TP (independent variable) as seen in Table 4. The canonical loadings represent the importance of each original variable to explain its respective canonical statistical variable. The loadings express the importance of each dependent or independent variable to derive the linear combinations between them. According to Hair Jr et al. [59], the higher the canonical loadings, the more important the variable to derive the canonical statistical variable. In this study, the traits LL, FCI, BW, BL, DHI, HEW, and TP were considered most important to derive the linear combinations between them. While Thomas and Chakravarty [7] related that FCI was also an important trait to improve reproductive efficiency. The high canonical loading of body morphometric BW, BL, DHI, HEW, and TP could imply that the combination of these traits is enough to explain the body size variation of buffalo. Our results also showed that MP, CI, TW, RW, RL and BD presented low canonical loadings, which many help in explaining of the relationship between both studied groups (Milk/reproduction and body morphometric traits), as shown in Fig 3 and Table 4.

The cross-loadings indicate the canonical correlations of each observed variable (dependent or independent) with opposite canonical statistical variable. In this study, the cross-loadings showed that milk/reproductive traits were correlated with body morphometric traits, confirming the relationship between both groups (Table 4). The higher cross-loadings for body morphometric were BW, BL, HEW, DHI, and TP, and for milk/reproductive traits were LL and FCI. The canonical correlations (cross-loadings) between body morphometric (BW, BL, HEW, DHI, and TP) and milk traits (LL) were negatives, suggesting that small size female buffalo may present higher LL. This might be due to a lower nutritional demand for the maintenance of small size, which could contribute to an increase in LL. Thus, larger body size would result in an increased nutritional demand for the maintenance of these animals, and consequently, the lower the energy available for LL. The canonical correlations (cross-loadings) between BW, BL, HEW, DHI, and TP and FCI were positive (Table 4), indicating that smaller-sized animals present lower FCI. This could be attributed to a lower requirement of maintenance energy by small females, and, thereby, early returning to ovarian activity. According to Mota et al. [60], larger animals present higher nutritional requirements for maintenance than smaller animals. In general, animals without enough nutrients would have impaired performance. Currently, research considering ideal body size in beef cattle has become relevant due to production and maintenance requirements. These factors influence the degree of physiological maturity of animals, and the economic return of the agribusiness [61]. Body size indicates important biological abilities related to adaptation, resistance, and type of exploration system. Research have shown that medium-sized animals are likely the most efficient biotype for the majority of production systems compared to small or very large animals [62,63]. However, the ideal biotype is controversial, and we must take into consideration the different production systems. In this study, the results show that female buffalo with lower BW, BL, DHI, HEW, and TP had lower FCI and higher LL. This information may aid selection programs, aiming to improve FCI and LL, as increasing these traits will not increase the body size of the female buffalo.

The squared loadings showed that the amount of shared explained variance by the dependent canonical statistical variable (milk and reproductive traits) was 14.51%. Among these, LL presented a higher percentage of explained variance (24.43%), followed by FCI (21.80%), confirming their importance among the dependent canonical statistical variables group. Whereas, the amount of shared variance in the body morphometric explained by the independent canonical statistical variable was 16.98%. The BW (61.99%), BL (31.76%), DHI (12.22%), HEW (14.98), and TP (25.51%), showed higher amounts of explained variance by the independent canonical statistical variables (Table 5). The results of the squared cross-loadings showed that approximately 7% of the total variance of LL and FCI can be explained by independent canonical statistical variables (body morphometric traits), as shown in Table 5. Regarding the independent variables (BW, BL, DHI, HEW, and TP), they presented higher shared explained variances by dependent canonical statistical variables, which were 19.92%, 10.20%, 3.93%, 4.78%, and 8.20%, respectively. These results could confirm the dependence between both groups of traits studied.

The CCA redundancy index showed the amount of explained variance between dependent canonical statistical variables (4.66%) and independent canonical statistical variables (5.45%), as shown in Table 5. CCA redundancy index quantifies the shared variance between canonical variables (dependent or independent). According to Hai Jr et al. [30], the CCA redundancy index should be considered to overcome the bias and uncertainty associated with the use of the canonical roots. In this study, the statistical index confirmed the dependency between both of the variables group, indicating that 4.66% of the milk/reproductive variance were explained by body morphometric, while 5.45% of the body morphometric variance were explained by milk/reproductive traits. In a study on Murrah buffalo, Thomas and Chakravarty [7] revealed a higher CCA redundancy index (47.69%) between breeding efficiency/breeding value for milk and growth/reproductive traits. The results of the sensitivity test showed that the elimination of any independent variable in the current CCA have not shown discrepancy between canonical loadings, cross-loadings, canonical correlations (r), canonical correlation coefficients (R^2^), p-values and CCA redundancy indexes (Table 6), thereby validating this CCA. Unfortunately, there have been few studies on the association between body traits and productive and reproductive systems in buffalo. The results of this study could provide valuable information for future designing of selection programs for buffalo breeding.

## Conclusions

Principal component analysis allows a reduction of the number of variables to explain the variance of the body conformation in crossbred progeny of Murrah × Jafarabadi buffalo. The body morphometric traits with higher contribution in body structural conformation were THW, HW, TW, LW, RW, HEW, BD, TP, RH, and BW, which accounted for almost half of the total variance. The canonical correlation analysis was efficient in explaining the dependence between milk/reproductive traits and body morphometry. The higher canonical correlation showed dependency between LL/CFI and BW, BL, DHI, HEW, and TP. These results suggest that female buffalo with smaller body size present higher LL and lower FCI. Thus, knowledge of the relationships between body morphometrics and their combinations with milk/reproductions traits could bring important contributions to indicate suitable functional type. Nevertheless, more research should be carried out taking into account the association between body morphometrics and productive/reproductive performance in the buffalo in order to better apply these relationships in breeding selection programs.

## Supporting information

S1 File This file contains information of the principal components (PC) calculation. (DOCX)

S2 File This file contains the Multicollinearity test—Variance inflation factor (VIFj). (DOCX)

S3 File This file contains table for weighting coefficients of last eight principal components and their body traits to explain the total variance in female Murrah × Jafarabadi buffalo. (DOCX)

S4 File This file shows the descriptive analyses for MP (Kg), LL (days), CI (days), FCI (days), and body morphometric traits (cm) of female Murrah × Jafarabadi buffalo. (DOCX)

S5 File This file contains the equations of linear combinations of the original variables and weighting coefficients of the first six principal components. (DOCX)

S6 File This file contains the eigenvectors of body morphometric traits on first, second and third principal components in female Murrah × Jafarabadi buffalo. (DOCX)

S7 File This file contains the eigenvalues (λi), percentages of described variance (DV), and accumulated explained variance (AEV) of milk/reproductive traits and body morphometric traits in female Murrah × Jafarabadi buffalo in four canonical functions (CF). (DOCX)

S1 Dataset This archive contains the data and information of animal studied. (XLS)
