# Supplementary material for: Multivariate analysis of body morphometric traits in conjunction with performance of reproduction and milk traits in crossbred progeny of Murrah × Jafarabadi buffalo (Bubalus bubalis) in North-Eastern Brazil
Source: PLoS One. 2020 Apr 21;15(4):e0231407. doi: 10.1371/journal.pone.0231407 (PMC7173789; doi:10.1371/journal.pone.0231407)
Supplement: S1 File — (DOCX) [file pone.0231407.s001.docx]

**S1 File**

**Calculation of principal components**

Principal components are calculated by linear combinations of the original variables with eigenvectors:

$Y_{i1}=a_{1}x_{i1}, a_{2}x_{i2}, \ldots, a_{p}x_{ip}$ $(1)$

If **Y**_i2_ is another principal component, it is a new linear combination:

$Y_{i2}=b_{1}x_{i1}, b_{2}x_{i2}, \ldots, b_{p}x_{ip}$ $(2)$

where the first principal component (**Y**_i1_) explains the highest percentage of the total variance, the second principal component (**Y**_i2_) explains the second most, and so on, until all the variance is explained. In a data set with p variables, the random vector X′ = [X_1_, X_2_, …, X*_p_*], having correlation matrix R with eigenvalue-eigenvector pairs (λ_i_, e_i_), for I = 1, 2, …, *p*, where λ_1_ ≥ λ_2_ ≥ … ≥ λ*_p_* ≥ 0 [37]. The _i_th principal component is given by

$PCi=e{}_{i}^{t}x= e_{i1}x_{1}+ e_{i2}x_{2}+\ldots+e_{ip}x_{p}$ $(3)$

where e*_ip_* is the *_p_*th eigenvector and x*_p_* is the *_p_*th value of the original variable.

Principal component analysis aimed to reduce the sample space disposal of variables associated with the PCs excluded. Criterion of Jolliffe [33,34] was applied to choose PC and discard of variables, Table 1 Supplement. To retain the PCs that explain the majority of total variation, this criterion consisted of the exclusion of the principal components with eigenvalues below 0.70 (λ < 0.7), because they contribute little to explaining the total variability. Variables with a greater weighting coefficient (in absolute value) will be discarded.
