# Supplementary material for: Multivariate analysis of body morphometric traits in conjunction with performance of reproduction and milk traits in crossbred progeny of Murrah × Jafarabadi buffalo (Bubalus bubalis) in North-Eastern Brazil
Source: PLoS One. 2020 Apr 21;15(4):e0231407. doi: 10.1371/journal.pone.0231407 (PMC7173789; doi:10.1371/journal.pone.0231407)
Supplement: S2 File — (DOCX) [file pone.0231407.s002.docx]

**S2 File**

**Multicollinearity test of the studied variables (variance inflation factor (VIF_j_).**

After the normality test of the data was verified, the multicollinearity between the variables was calculated by test of variance inflation factor (VIF_j_), Table1, according to following:

$${VIF}_{j}=\frac{1}{1-R_{j}^{2}} (1)$$

where $R_{j}^{2}$ presents the partial determination coefficient of $\boldsymbol{X}_{j}$ in relation to the other exploratory variables, **j** = 1, 2, 3, …, p (number of predictive variables). Variance inflation factors greater than 10 (VIF > 10) indicate that there is multicollinearity in any set of variables $\boldsymbol{X}_{j}$. In this case, it also indicates how the variation of an estimator is inflated because of the multicollinearity. The larger the multicollinearity, the lower the ability to identify the effects of the variables in CCA, making the interpretation less reliable [37].

**S2 Table 1. Variance inflation Factor (VIF) for milk/reproductive and body morphometric traits in crossbred progeny of Murrah × Jafarabadi buffalo.**

| Variables^1^ | VIF |
| --- | --- |
| Milk/reproductive traits |  |
| MP | 1.5408 |
| LL | 1.5966 |
| CI | 1.6381 |
| FCI | 1.7418 |
| Body morphometric traits |  |
| BW | 1.8734 |
| TW | 1.3621 |
| RW | 1.3472 |
| RL | 1.3886 |
| BD | 2.1120 |
| BL | 1.6710 |
| DHI | 1.2898 |
| HEW | 1.6717 |
| TP | 1.7906 |

^1^Milk production (MP), lactation length (LL), calving interval (CI), first calving interval (FCI), breast width (BW), thorax width (TW), rump width (RW), rump length (RL), body depth (BD), body length (BL), distance from the head to the ischium (DHI), height of withers (HEW), and thoracic perimeter (TP).
