# Supplementary material for: Multivariate analysis of body morphometric traits in conjunction with performance of reproduction and milk traits in crossbred progeny of Murrah × Jafarabadi buffalo (Bubalus bubalis) in North-Eastern Brazil
Source: PLoS One. 2020 Apr 21;15(4):e0231407. doi: 10.1371/journal.pone.0231407 (PMC7173789; doi:10.1371/journal.pone.0231407)
Supplement: S3 File — (DOCX) [file pone.0231407.s003.docx]

| Traits^1^ | *µ* | *s* | Min | Max |
| --- | --- | --- | --- | --- |
| Milk/reproductive |  |  |  |  |
| MP | 1,656.00 | 609.32 | 701.00 | 3,403.00 |
| LL | 257.20 | 56.05 | 135.00 | 449.00 |
| CI | 441.10 | 47.54 | 357.30 | 561.00 |
| FCI | 489.20 | 117.00 | 334.00 | 930.00 |
| Body morphometric traits |  |  |  |  |
| BW | 43.72 | 5.21 | 32.00 | 54.00 |
| SW | 33.20 | 4.44 | 14.00 | 42.00 |
| TW | 35.38 | 4.43 | 25.00 | 49.00 |
| LW | 37.72 | 4.75 | 28.00 | 52.00 |
| THW | 51.43 | 5.39 | 40.00 | 63.00 |
| HW | 40.03 | 5.61 | 25.00 | 56.00 |
| RW | 25.44 | 4.21 | 17.00 | 38.00 |
| RL | 39.86 | 4.06 | 32.00 | 56.00 |
| BD | 71.77 | 6.25 | 55.00 | 88.00 |
| BL | 143.33 | 4.78 | 120.00 | 160.00 |
| DHI | 182.80 | 9.40 | 146.00 | 204.00 |
| HEW | 130.50 | 5.03 | 116.00 | 148.00 |
| RH | 134.00 | 4.78 | 119.00 | 143.00 |
| TP | 201.30 | 8.10 | 180.00 | 223.00 |

**S3 File**

**S3 Table**. **Descriptive analyses for MP (Kg), LL (days), CI (days), FCI (days), and body morphometric traits (cm) in crossbred progeny of Murrah × Jafarabadi buffalo.**

Average (µ), standard deviation (s), maximum values (Max), minimum values (Min). ^1^Milk production (MP), lactation length (LL), calving interval (CI), first calving interval (FCI), breast width (BW), shoulder width (SW), thoracic width (TW), loin width (LW), thigh width (THW), hip width (HW), rump width (RW), rump length (RL), body depth (BD), body length (BL), distance from the head to the ischium (DHI), height withers (HEW), rear height (RH), and thoracic perimeter (TP).
