# Supplementary material for: Multivariate analysis of body morphometric traits in conjunction with performance of reproduction and milk traits in crossbred progeny of Murrah × Jafarabadi buffalo (Bubalus bubalis) in North-Eastern Brazil
Source: PLoS One. 2020 Apr 21;15(4):e0231407. doi: 10.1371/journal.pone.0231407 (PMC7173789; doi:10.1371/journal.pone.0231407)
Supplement: S4 File — (DOCX) [file pone.0231407.s004.docx]

**S4 File**

**S4 Table. Weighting coefficients of the last eight principal components (PC) and their body morphometric traits to explain the variance in crossbred progeny of Murrah × Jafarabadi buffalo.**

| Traits^1^ | PC7 | PC8 | PC9 | PC10 | PC11 | PC12 | PC13 | PC14 |
| --- | --- | --- | --- | --- | --- | --- | --- | --- |
| BW | -0.2628 | -0.3693 | 0.2665 | -0.4844 | -0.0190 | -0.0153 | -0.0982 | 0.0925 |
| THW | 0.0214 | 0.3182 | 0.2096 | -0.1245 | 0.1727 | 0.1986 | 0.4870 | 0.2530 |
| **HW** | -0.1210 | 0.0772 | -0.1385 | -0.2006 | -0.1160 | -0.1278 | **-0.6637** | -0.2047 |
| **RW** | 0.0745 | **-0.7284** | -0.2022 | 0.0111 | 0.3190 | 0.0624 | 0.1037 | 0.1443 |
| **RL** | -0.1453 | -0.0752 | 0.0931 | 0.0465 | -0.3986 | -0.4030 | 0.0751 | **0.5602** |
| BD | -0.2444 | 0.0696 | -0.3298 | 0.0094 | -0.1623 | 0.0285 | 0.3733 | -0.5017 |
| BL | 0.2356 | 0.1293 | 0.4238 | 0.0573 | 0.1985 | 0.4264 | -0.3132 | 0.0691 |
| HEW | -0.2204 | 0.3388 | -0.1065 | 0.0517 | -0.0687 | 0.0121 | -0.1355 | 0.2900 |
| **RH** | 0.1655 | -0.0731 | **0.4855** | -0.3467 | -0.2383 | -0.0550 | 0.1631 | -0.3911 |
| SW | -0.5432 | -0.0529 | 0.0636 | 0.2657 | 0.3410 | 0.2523 | -0.0820 | -0.0670 |
| **TW** | **0.5870** | -0.0059 | -0.2900 | -0.0160 | 0.0302 | 0.0368 | -0.0725 | 0.0793 |
| **LW** | 0.1032 | 0.2340 | -0.2755 | -0.4333 | **0.4262** | -0.2654 | 0.0171 | 0.0277 |
| **DHI** | 0.1105 | -0.1257 | -0.2142 | 0.0616 | -0.5141 | **0.5136** | -0.0240 | 0.0647 |
| **TP** | 0.1881 | -0.0710 | 0.2662 | **0.5645** | 0.0733 | -0.4390 | -0.0318 | -0.2041 |

^1^Breast width (BW), thigh width (THW), hip width (HW), rump width (RW), rump length (RL), body depth (BD), body length (BL), height withers (HEW), rear height (RH), shoulder width (SW), thoracic width (TW), loin width (LW), distance from the head to ischium (DHI), and thoracic perimeter (TP).
