# Supplementary material for: Multivariate analysis of body morphometric traits in conjunction with performance of reproduction and milk traits in crossbred progeny of Murrah × Jafarabadi buffalo (Bubalus bubalis) in North-Eastern Brazil
Source: PLoS One. 2020 Apr 21;15(4):e0231407. doi: 10.1371/journal.pone.0231407 (PMC7173789; doi:10.1371/journal.pone.0231407)
Supplement: S5 File — (DOCX) [file pone.0231407.s005.docx]

**S5 File**

**S5 File. Equations of linear combinations of the original variables and weighting coefficients of the first six PCs.**

$$Y_{1}=-0.38THW-0.34HW-0.32TW-0.32LW-0.30RW-0.29HEW-0.29BD-0.25SW-0.24BL-0.23TP-0.21RH-0.17RL+0.12BW-0.12DHI \boldsymbol{(1)}$$

$$Y_{2}=0.19THW+0.13HW+0.00TW+0.23LW+0.23RW-0.27HEW+0.18BD-0.23SW-0.11BL-0.48TP-0.48RH+0.07RL-0.45BW-0.06DHI \boldsymbol{(2)}$$

$$Y_{3}=0.00THW-0.12HW-0.42TW-0.12LW-0.11RW+0.16HEW+0.38BD-0.43SW+0.38BL-0.04TP+0.04RH+0.40RL+0.04BW+0.33DHI \boldsymbol{(3)}$$

$$Y_{4}=-0.21THW-0.34HW-0.01TW+0.25LW-0.14RW+0.24HEW+0.22BD-0.05SW-0.19BL+0.05TP+0.28RH+0.25RL-0.35BW-0.58DHI \boldsymbol{(4)}$$

$$Y_{5}=-0.15THW-0.29HW+0.36TW+0.11LW-0.48RW-0.35HEW+0.24BD+0.40SW+0.21BL-0.05TP-0.11RH+0.11RL-0.14BW-0.29DHI \boldsymbol{(5)}$$

$$Y_{6}=-0.27THW+0.25HW-0.02TW+0.20LW+0.10RW-0.23HEW-0.07BD+0.07SW-0.37BL+0.20TP-0.15RH+0.67RL+0.32BW+0.05DHI \boldsymbol{(6)}$$
