# Supplementary material for: Multivariate analysis of body morphometric traits in conjunction with performance of reproduction and milk traits in crossbred progeny of Murrah × Jafarabadi buffalo (Bubalus bubalis) in North-Eastern Brazil
Source: PLoS One. 2020 Apr 21;15(4):e0231407. doi: 10.1371/journal.pone.0231407 (PMC7173789; doi:10.1371/journal.pone.0231407)
Supplement: S6 File — (DOCX) [file pone.0231407.s006.docx]

**S6 File**

Figures of eigenvectors of body measurements on first, second and third principal components in crossbred progeny of Murrah × Jafarabadi buffalo.


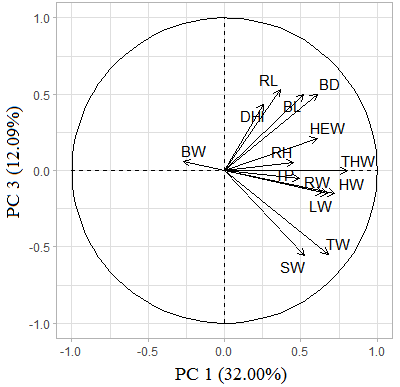


**S6 Fig 1.** **Eigenvectors of body measurements on first and third principal components in crossbred progeny of Murrah** × **Jafarabadi buffalo.** Thigh width (THW), hip width (HW), thoracic width (TW), loin width (LW), rump width (RW), height withers (HEW), body depth (BD), shoulder width (SW), body length (BL), thoracic perimeter (TP), rear height (RH), rump length (RL), breast width (BW), and distance from the head to ischium (DHI).


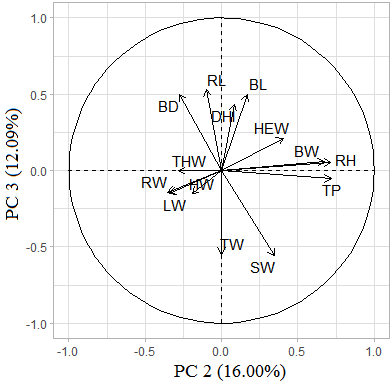


**S6 Fig 2. Eigenvectors of body measurements on second and third principal components in crossbred progeny of Murrah** × **Jafarabadi buffalo.** Thigh width (THW), hip width (HW), thoracic width (TW), loin width (LW), rump width (RW), height withers (HEW), body depth (BD), shoulder width (SW), body length (BL), thoracic perimeter (TP), rear height (RH), rump length (RL), breast width (BW), and distance from the head to ischium (DHI).
