# Supplementary material for: Multivariate analysis of body morphometric traits in conjunction with performance of reproduction and milk traits in crossbred progeny of Murrah × Jafarabadi buffalo (Bubalus bubalis) in North-Eastern Brazil
Source: PLoS One. 2020 Apr 21;15(4):e0231407. doi: 10.1371/journal.pone.0231407 (PMC7173789; doi:10.1371/journal.pone.0231407)
Supplement: S7 File — (DOCX) [file pone.0231407.s007.docx]

**S7 File**

**S7 Table. Canonical functions (CF), eigenvalues (λ_i_), percentages of described variance (DV, and accumulated explained variance (AEV) of milk/reproductive and body morphometric traits in crossbred progeny of Murrah × Jafarabadi buffalo**.

| CF | λ_i_ | (%) DV | (%) AEV |
| --- | --- | --- | --- |
| 1 | 0.4734 | 72.458 | 72.46 |
| 2 | 0.0900 | 13.781 | 86.24 |
| 3 | 0.0570 | 8.727 | 94.97 |
| 4 | 0.0329 | 5.033 | 100.00 |
